# Synthesis and Characterizations of (Co*_x_*Mg_(2−*x*)_)SiO_4_ Forsterite Ceramic Pigments from Mirror Waste

**DOI:** 10.3390/ma11071210

**Published:** 2018-07-13

**Authors:** Niti Yongvanich, Kullada Supanichwatin, Jitat Penglan, Narit Triamnak

**Affiliations:** Department of Materials Science and Engineering, Faculty of Engineering and Industrial Technology, Silpakorn University, Nakhon Pathom 73000, Thailand; niti.yongvanich@gmail.com (N.Y.); kang.kullada@gmail.com (K.S.); chaingather_kub@hotmail.com (J.P.)

**Keywords:** ceramic pigment, forsterite, mirror waste

## Abstract

Ceramic pigments have been widely used in a variety of industries because of their excellent properties, such as high thermal stability, low-cost productions, and simple manufacturing processes. Re-use of mirror waste, which consists of silicon dioxide greater than 70%, is a method that can reduce raw materials cost. In this work, ceramic pigment with forsterite structure, Mg_2_SiO_4_, was synthesized via conventional solid state reaction by using mirror waste as a precursor. Solid solutions of Co-doped forsterite pigment, Co*_x_*Mg_(2−*x*)_SiO_4_ where *x* = 0.02–1.6, were calcined at 1000 °C for 2 h. The calcined powders were characterized by X-ray diffraction technique (XRD), Fourier-transform infrared spectroscopy (FTIR), scanning electron microscope (SEM), X-ray photoelectron spectroscopy (XPS), UV-Vis spectrophotometer, and color measurement (CIEL*a*b*). XRD results confirmed that forsterite phase was found as a main phase in the ceramic powder. However, the forsterite phase decreased with increased concentration of Co to *x* = 0.8–1.6. This could be because of the solubility limit of Co in solid solution. In addition, the use of mirror waste as a raw material was able to reduce calcination temperature compared to the use of oxide reagents. Color measurements or CIEL*a*b* color space of forsterite pigments were located in red-blue quadrant for Co-doped pigment.

## 1. Introduction

Ceramic pigments have been used extensively in decorative industries as a coloration component due to their advantages such as low-cost productions, stability, longevity, and mass-producibility. Even though ceramic pigments possess many advantages, the reduction of raw materials cost is still needed to compete in the market. Re-use of industrial waste is the current trend to lower the cost of raw materials. Traditionally, this waste is disposed of in landfill. It includes glass and mirror waste. The advantage of these wastes for re-use is their amorphous structure. Such structure could lead to a lower reaction temperature. However, this might cause a variation in pigment properties, such as firing formula and color tone, due to the composition differences between the waste and pure reagents. In addition, this variation could be seen between wastes of similar chemical compositions, such as glass and mirror, as well. Even though the secondary processing of glass is common, the aluminum film in mirror waste might deviate the final product properties. Practically, if the desirable pigment phase can be obtained as well as excellent chromatic quality in term of both stability and color tone, then the waste can be used. This method could be optimized and could lead to benefits both economically and environmentally.

Generally, the pigments are used as a dispersed phase in media such as glaze, ceramic body, and porcelain enamels causing the desirable color in applications. Such applications usually involve high-temperature processes. Therefore, the synthesized pigments must exhibit excellent thermal and chemical stability. There have been researches into the synthesis, phase formation, and thermal stability in Co olivines and related compounds such as CaCoSi_2_O_6_, Ca_2_CoSi_2_O_7_, and CaCoSiO_4_ [1,2,3]. Forsterite [4,5], with the chemical formula Mg_2_SiO_4_, is a crystalline magnesium silicate and seems to be usable as the ceramic pigment due to its fabulous refractoriness. It shows high thermal stability with a melting temperature of 1890 °C as well as high chemical stability. In contrast, the common way to synthesize forsterite also requires very high temperatures. It is usually fired at a temperature of approximately 1500 °C. Therefore, it is crucial to lower the synthesized temperature which also means lowering productions cost. In addition, the thermal stability of forsterite pigment needs to be investigated simultaneously to understand and optimize the pigment preparation processes.

This study aims to synthesize the forsterite pigment and characterize its properties by using mirror waste as a raw material and cobalt oxide as a chromophore of the pigment. Phase evolution and chromatic properties of the pigment are investigated with the different chromophore contents.

## 2. Materials and Methods

Forsterite powder was prepared via a conventional solid state reaction method by stoichiometrically mixing reagent-grade silicon dioxide (SiO_2_) and magnesium oxide (MgO) as precursors. The mixture was wet ball-milled for 24 h with ethanol followed by calcination in an alumina crucible at temperatures between 800 and 1200 °C for 2 h. The as-calcined powder was then ground in a mortar prior to structural characterization by X-ray diffraction technique (XRD, LabX XRD-6100 Diffractometer, Shimadzu, Kyoto, Japan) with Cu-Kα radiation at λ = 1.5406 Å. Cobalt-doped forsterite pigment (Co*_x_*Mg_(2−*x*)_SiO_4_, when *x* = 0–1.6) was synthesized by mixing mirror waste (as the SiO_2_ source) instead of reagent-grade SiO_2_, MgO and cobalt oxide (Co_2_O_3_), stoichiometrically. The mixed powder was subjected to the previous preparation method as the reagent grade forsterite powder. Phase evolution of the pigment powder from mirror waste was comparatively investigated by XRD technique.

Fourier transformation infrared spectroscopy (FTIR, Vertex70, Bruker, Billerica, MA, USA) was performed as a complement study for pigment structural investigation. 

X-ray fluorescence (XRF, Minipal-4, PANalytical, Almelo, The Netherlands) was used to characterize mirror waste composition, as shown in Table 1. The morphology and elemental distribution of the powder were examined by scanning electron microscope (SEM, TM3030, Hitachi, Tokyo, Japan) and energy dispersive spectroscopy (EDS, TM3030, Hitachi). 

Optical properties of the pigment were investigated by using the Uv-Vis spectroscopy technique (UV-1800, Shimadzu) to study light reflection character and the CIELab method by colorimeter (Color Reader CR-10, Minolta, Osaka, Japan) was used to examine chromatic values. The color is defined as the three parameters, L*, a* and b*, where they represent the brightness, change from green (negative value) to red (positive value), and change from blue (negative value) to yellow (positive value), respectively.

## 3. Results and Discussion

**Figure 1:**
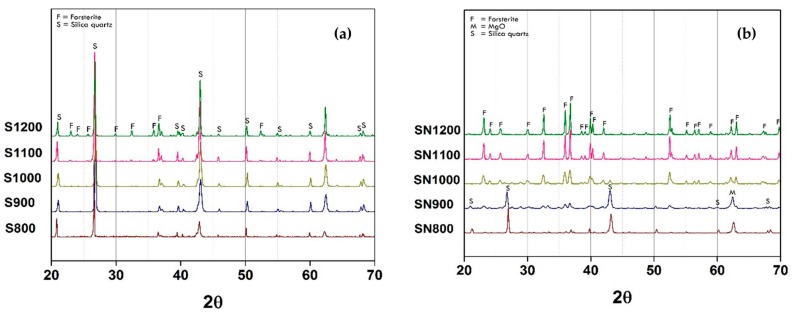
X-ray diffraction (XRD) patterns of as-calcined forsterite powder at various temperatures between 800 and 1200 °C by mixing reagent-grade SiO_2_: (**a**) Without NaF mineralizer; (**b**) Added 5 wt. % of NaF.

The possibility of using mirror waste to synthesize forsterite pigment via the common route preparation, such as using reagent-grade SiO_2_ as the precursor, needed to be investigated. Phase evolution of this composition is illustrated in Figure 1.

It is clearly shown in Figure 1a that the forsterite phase could not be observed even if the calcination temperature was increased to 1000 °C. With further temperature increase to 1200 °C, there was a small peak pattern of forsterite phase occurrence. On the other hand, the main phase of this powder remained as SiO_2_ (labeled with “S”) which identified with JCPDF 00-046-1045. This results confirmed the difficulty of forsterite synthesis at moderate temperature by conventional solid state reaction because of its excellent refractoriness [6]. Therefore, the addition of a mineralizer, NaF, was performed to increase the raw materials diffusion rate leading to a reduction of the reaction temperature. The XRD pattern of NaF-added powder is shown in Figure 1b. Forsterite structure peaks (labeled with “F”) were observed with small intensity when the powder was calcined at temperatures as low as 800 °C which was matched to JCPDF 00-034-0189. The peak pattern intensity increased clearly when the temperature increased to 1000 °C. In addition, there was an abrupt decreased of SiO_2_ precursor peaks. The single phase of forsterite structure was obtained when the calcination temperature increased up to 1100 °C. According to data in Table 1, the second highest elemental concentration of mirror waste was sodium which could play a role in forsterite formation as a similar mineralizer, NaF [6,7,8,9].

**Figure 2:**
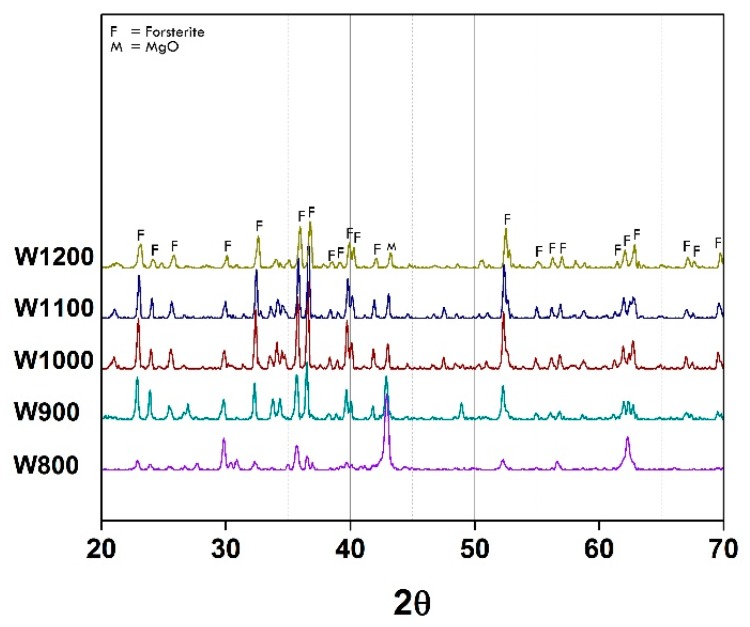
XRD patterns of as-calcined forsterite powder from mirror waste at various temperatures between 800 and 1200 °C.

XRD pattern of as-calcined powder Co*_x_*Mg_(2−*x*)_SiO_4_, when *x =* 0, synthesized from mirror waste are shown in Figure 2. The forsterite peaks can be clearly seen when the powder was calcined at the temperature of 800 °C, and their intensity significantly increased with the calcination temperature increase. It was observed that using mirror waste as the SiO_2_ source could reduce the forsterite formation temperature [10]. This could be because of the disorder-structure within mirror waste from industrial post-processes. In addition, other oxides components, especially Na_2_O, could behave as the mineralizer. It needs to be noted that the secondary phases appeared in all preparation conditions. This is because of the high impurities content in mirror waste which is very difficult to eliminate and control. However, compared to the main phase, the number of secondary phases appears to be small and influences on the pigment properties are correspondingly small.

**Figure 3:**
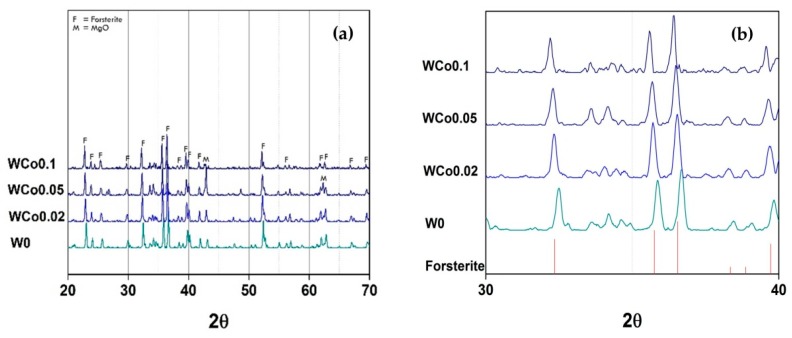

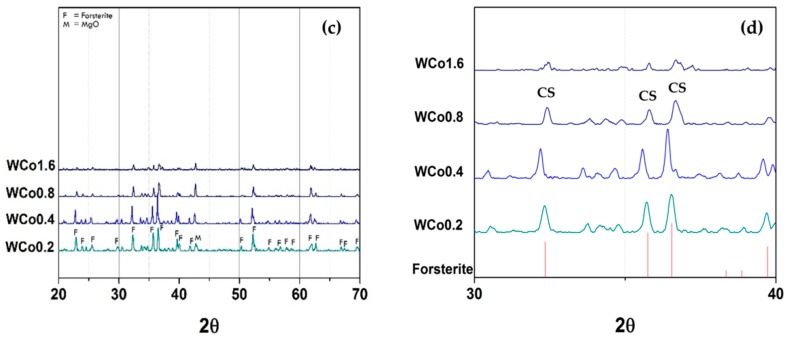
XRD patterns of Co*_x_*Mg_(2−*x*)_SiO_4_ sample synthesized by mirror waste calcined at 1000 °C for 2 h: (**a**,**b**) *x =* 0–0.1; (**c**,**d**) *x =* 0.2–1.6.

The crystal structure characterization of forsterite mirror waste pigment with increasing concentration of Co chromophore is depicted in Figure 3. This investigation was designed to study structural stability based on the content of chromophore ion. The concentration of Co ion was varied, *x =* 0–1.6, with a constant calcination temperature of 1000 °C. This temperature was enough to obtain forsterite phase in the pigment as noted in previous XRD results. It was found that the increase of Co ion content from *x =* 0 to *x =* 0.4 caused the shifting of the peaks pattern towards a smaller diffraction angle. It could be implied that the d-spacing of unit cell increased because of the greater Co ion diffusion into the structure. The changes of unit cell volume with different Co content were observed in related compounds such as CaCoSi_2_O_6_ pyroxenes [11,12] as well. However, when the content of Co was increased to *x =* 0.8–1.6, the XRD patterns exhibit the Co_2_SiO_4_ phase which matched to JCPDF 00-015-0865, labeled “CS” in Figure 3d. In addition, peak intensity of this phase reduced. It might be because the excess Co interacted with the other oxides within the mixture resulting in an increase of secondary phases and decrease of the main phase [8,13]. 

**Figure 4:**
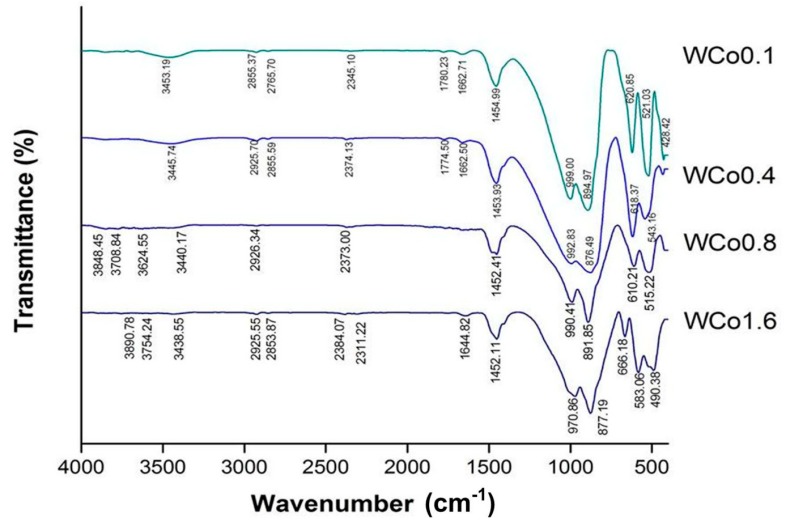
Fourier transformation infrared spectroscopy (FTIR) Spectrum of Co*_x_*Mg_(2−*x*)_SiO_4_ (*x =* 0.1, 0.4, 0.8, 1.6) samples synthesized by mirror waste calcined at 1000 °C for 2 h.

FTIR was studied as the supplementary structural characterization and is illustrated in Figure 4. The results exhibited small peaks at wave number 2800–3600 cm^−1^ which refer to the vibration of O-H stretching mode. These peaks were probably a result of humidity that KBr absorbed while the measurement was being conducted. In addition, the peak of O-H bending was observed at wave number of 1454 cm^−1^ which is also the evidence of water molecule existence. After that, peaks of Si-O bonding were seen at 894 and 999 cm^−1^ which are Si-O-Si stretching [14,15]. Finally, Si-O-Si bending peaks were observed at 521 and 620 cm^−1^. As the content of Co ion increased both Si-O-Si stretching and bending peaks broadened [16]. The appearance of peak broadening was evidence of structural distortion because of the diffusion of Co ion in the structure. These results are in agreement with XRD data.

**Figure 5:**
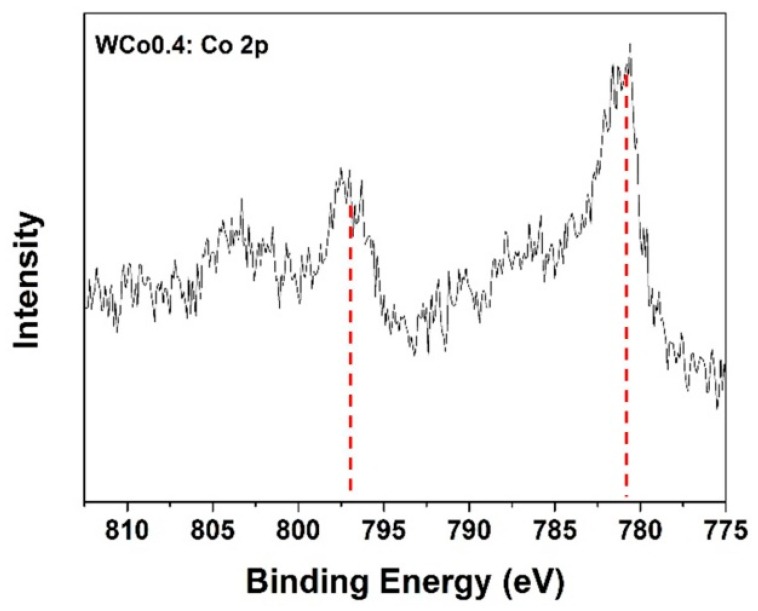
X-ray photoelectron spectroscopy (XPS) spectra of Co_0.4_Mg_1.6_SiO_4_ samples showing binding energy of Co 2p.

To predict the behavior of Co ion, Co_0.4_Mg_1.6_SiO_4_ powder was then analyzed by X-ray photoelectron spectroscopy technique (XPS). The result of Co-2p binding energy is shown in Figure 5. It was found that there were peaks split into Co-2p_1/2_ and Co-2p_3/2_ at 797 eV and 781 eV, respectively. This result described the existence of divalent cobalt (Co^2+^) which could mean the substitution of Co ion at the Mg position in the forsterite structure. Characterized data from XRD, FTIR, and XPS confirm that Co-doped forsterite pigment was successfully synthesized by using mirror waste as the SiO_2_ source.

**Figure 6:**
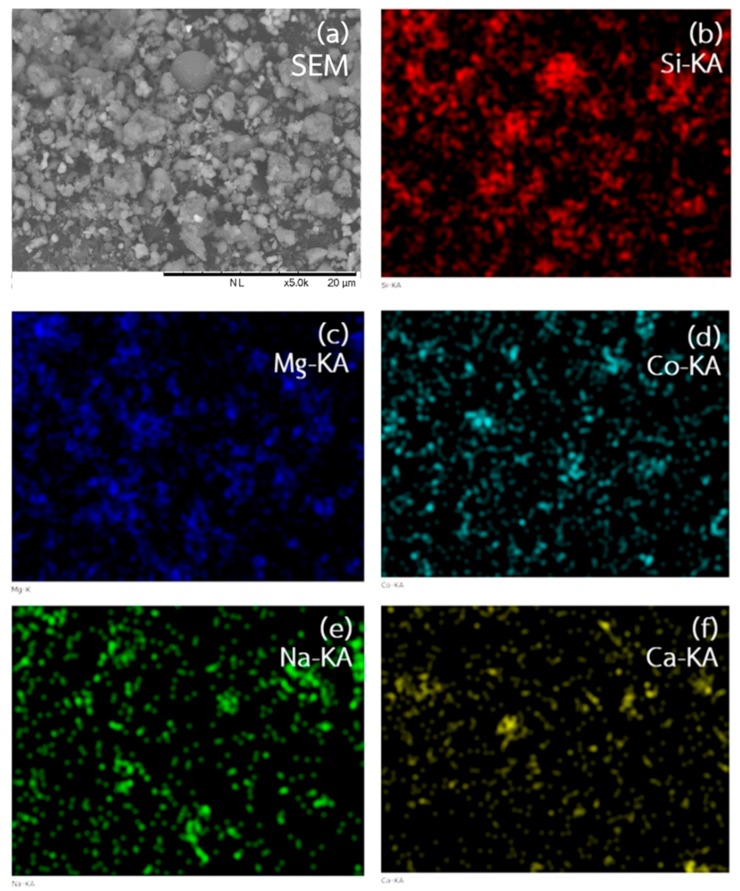
Scanning electron microscope (SEM) micrograph and energy dispersive spectroscopy (EDS) data of Co_0.4_Mg_1.6_SiO_4_ powder: (**a**) morphology; (**b**) silicon; (**c**) magnesium; (**d**) cobalt; (**e**) sodium; and (**f**) calcium.

The morphology and elemental distribution of the Co_0.4_Mg_1.6_SiO_4_ powder obtained from SEM and EDS techniques are shown in Figure 6. It was found that the particle shape of the pigment was irregular. In addition, particle size was non-uniform. It could be a result of the non-uniformity of mirror waste structure. The majority elements of the pigment were Si and Mg as expected. Moreover, the other elements such as Co, Na and Ca were observed with small concentrations which agreed with XRF data listed in Table 1. However, distribution of Co, which was doped into the forsterite pigment, was uniform.

**Figure 7:**
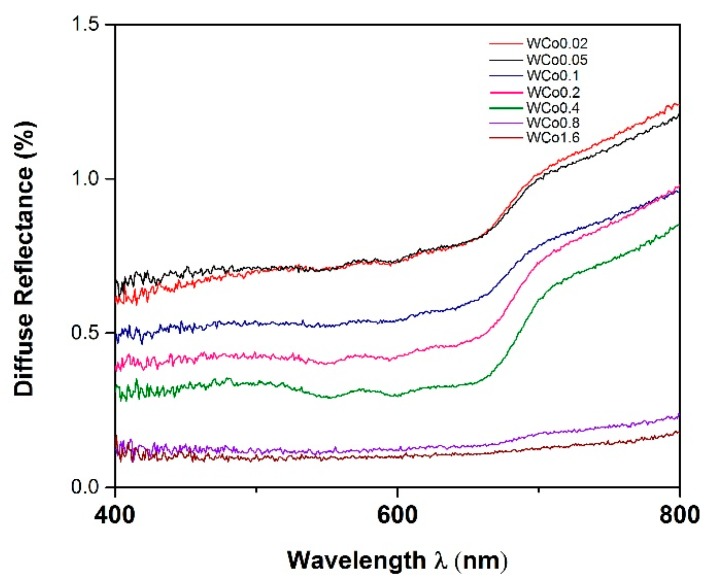
Diffuse reflectance spectra of Co*_x_*Mg_(__2−*x*)_SiO_4_ (where *x* = 0.02–1.6) synthesized by mirror waste calcined at 1000 °C for 2 h.

**Figure 8:**
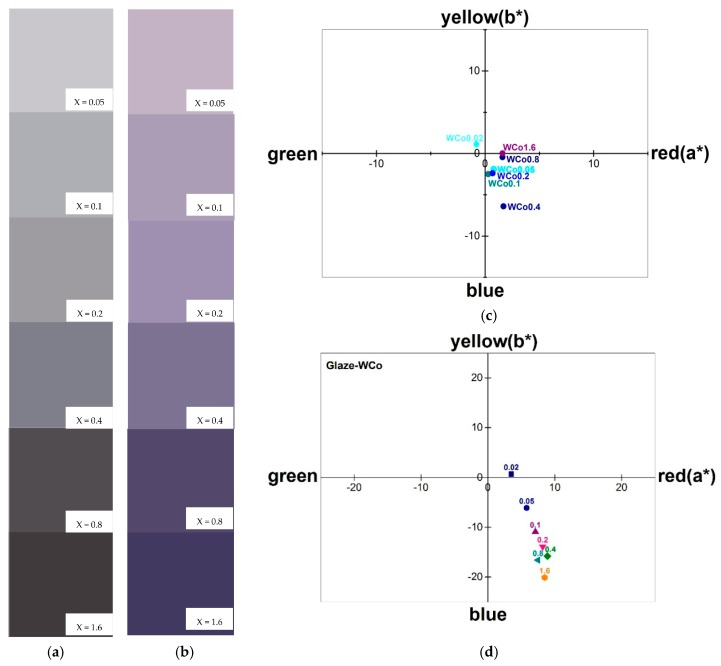
Chromatic tone and CIEL*a*b* diagram of Co*_x_*Mg_(2−*x*)_SiO_4_ (where *x =* 0.05–1.6): (**a**,**c**) As-calcined powder; (**b**,**d**) Mixing glaze fired powder.

UV-Vis spectroscopy spectra are illustrated in Figure 7. It was found that the absorption spectra were at the wavelength of 550–700 nm. Such absorbed wavelength range is due to the excitation of electron from 4T1(F) to 4T1(P) and 2T1(G) to 2T2(G) states of Co^2+^ chromophore which located at the octahedral sites of the forsterite structure [17,18]. There are three absorption peaks in the 550–700 nm range (absorbing yellow, orange, and red). The observed color, according to the complimentary color theory, is mainly green and blue (430–560 nm) which was clearly reflected by dramatic changes in both a and b color parameters in Table 2. The a parameter remained in the redness regime whereas the b parameter displayed blueness. Upon increasing the amount of cobalt doping, slight increases in the intensity of the absorption peaks were observed as the peaks became more prominent. This phenomenon was also confirmed by an enhancement of more than double for both a and b parameters when increasing cobalt concentration from *x =* 0.2 to *x =* 0.4. This result is likely to be associated with the absence of free cobalt oxide observed by XRD and the XRD peak shift as a result of successful cobalt incorporation into the Mg lattice. As the content of Co increased to x *=* 0.8–1.6, absorption strongly appeared through the full range of measured wavelength leading to dark gray color which is shown in Figure 8 [17].

**Figure 9:**
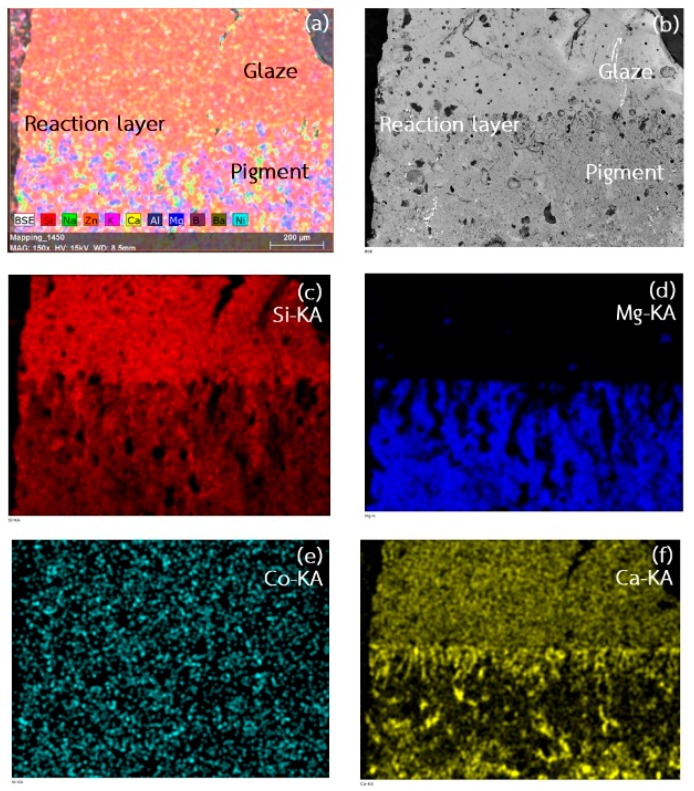
EDS mapping of the interface between Co_0.1_Mg_1.9_SiO_4_ pigment and low-temperature glaze: (**a**) existing elements; (**b**) morphology of inter-layer between pigment and glaze; (**c**) silicon; (**d**) magnesium; (**e**) cobalt; and (**f**) calcium.

Chromatic characteristics of Co*_x_*Mg_(2−*x*)_SiO_4_ with various content of Co are illustrated in Figure 8. Parameters L*, a* and b*, which were measured by CIELab method, are listed in Table 2. Brightness, L*, decreased as the Co content increased leading to a darker tone and was seen in the similar trend of UV-Vis spectra. In addition, Co*_x_*Mg_(2−*x*)_SiO_4_ pigments tended to exhibit their color in positive a* and negative b* coordination as shown in Figure 8b which showed violetish color [19,20]. As pigment application is generally dispersed in glaze, its glazed chromatic properties were investigated and are shown in Figure 8b,d. Pigments were mixed with low-temperature glaze powder (1000 °C firing glaze) with 10:90 wt. % ratio and followed by firing at 1000 °C for 2 h. After firing, the mixtures were ground to a fine powder before color measurement. It was found that the color of the mixtures changed and exhibited more violetish than as-calcined pigments as shown in Figure 8a,c. The parameters a* and b* showed a greater value in the identical quadrantal coordination as shown in Figure 8d. The differences in pigment color quality are listed in Table 3 which are calculated by an equation as follows:

(1) $$dE^{*}=\sqrt{dL^{*2}+da^{*2}+db^{*2}}$$

where dE* identified as the color stability of the pigments after glaze which is calculated from the differences of the L*, a*, and b* parameters from prior to and after being glazed. Generally, the acceptable value of dE* should be less than 1. It was discovered that the calculated value of the pigments was greater than 1 in every doping compositions, shown in Table 3. The unsteady color changes also tend to increase as the Co content increased. It is probably because of the excess Co ions’ outward diffusion from the forsterite pigments. This prediction was confirmed by the EDS results which are depicted in Figure 9. The interface between as-calcined powder and low-temperature glaze powder (CG466) was created by cold isostatic compression to pellet form. Then it was subjected to the identical temperature profile as previous procedures. The elements existences at the interfacial vicinity were then investigated by EDS technique and are illustrated in Figure 9. It is clearly seen that Co ion diffused and leaked from the pigment region and spread homogeneously across the interface. The ready dissolution of Co was the origin of color changes of the pigments and exhibited insufficient stability within the glaze. This instability might be improved by further investigations, such as temperature profile adjustment in processing or co-dopings into the pigments. 

## 4. Conclusions

The pigments with Co-doped forsteritre structure were successfully synthesized from mirror waste. The phase evolution of the pigments was investigated, and it was found that mirror waste could decrease the calcination temperature. It was because of the other oxides within mirror waste, such as Na_2_O. FTIR and XPS measurements confirmed the existence of Co^2+^ ion in the pigment structure. The Co doping caused the forsterite pigment to color violetish. Color tone was darker as the concentration of doping ion increased which was illustrated by UV-Vis spectroscopy and the CIELab method. However, the forsterite pigments showed low stability in glaze which was because of the diffusion of Co chromophore. The stability of this mirror waste pigment could be enhanced by further investigation.

## Figures and Tables

**Table 1:** Compositions of oxides from mirror waste.

Oxides	Mol %
**SiO_2_**	71.11
**Na_2_O**	12.68
**CaO**	11.38
**MgO**	3.52
**Etc.**	1.31

**Table 2:** CIEL*a*b* data of as-calcined Co*_x_*Mg_(__2−*x*)_SiO_4_ (where *x* = 0.05–1.6) powder.

Co*_x_*Mg_(__2−*x*)_ SiO_4_ (*x*)	L*	a*	b*
0.05	79.9	0.8	−1.9
0.1	71.1	0.3	−2.5
0.2	64	0.7	−2.4
0.4	52.9	1.7	−6.4
0.8	32.8	1.6	−1.8
1.6	25.5	1.6	−0.1

**Table 3:** Color stability *d*E*of Co*_x_*Mg_(2−*x*)_SiO_4_ (where *x* = 0.05–1.6) pigments after being glazed.

Co*_x_*Mg_(__2−*x*)_SiO_4_ (*x*)	*d*E*
0.05	8.6
0.1	11.8
0.2	14.1
0.4	12.4
0.8	15.9
1.6	21.3
